# Diagnostic Value of a Rec-ELISA Using *Toxoplasma gondii* Recombinant SporoSAG, BAG1, and GRA1 Proteins in Murine Models Infected Orally with Tissue Cysts and Oocysts

**DOI:** 10.1371/journal.pone.0108329

**Published:** 2014-09-30

**Authors:** Mert Döşkaya, Ayşe Caner, Hüseyin Can, Sultan Gülçe İz, Yaprak Gedik, Aysu Değirmenci Döşkaya, Mina Kalantari-Dehaghi, Yüksel Gürüz

**Affiliations:** 1 Department of Parasitology, Ege University Medical School, Bornova/İzmir, Turkey; 2 Department of Molecular Biology, Ege University Faculty of Sciences, Bornova/İzmir, Turkey; 3 Department of Bioengineering, Ege University Faculty of Engineering, Bornova/İzmir, Turkey; 4 Department of Dermatology, University of California Irvine, Irvine, California, United States of America; Tulane University, United States of America

## Abstract

*Toxoplasma gondii* causes congenital toxoplasmosis in newborns resulting with fetal anomalies. Determining the initiation time of infection is very important for pregnant women and current serological assays have drawbacks in distinguishing the recently acute toxoplasmosis. Diagnosis of recently acute infection may be improved by using stage specific antigens in serological assays. In the present study, the diagnostic value of sporozoite specific SporoSAG, bradyzoite specific BAG1 proteins and GRA1 protein expressed by all forms of the parasite have been evaluated ELISA using sera systematically collected from mice administered orally with tissue cyst and oocysts. The *anti-*SporoSAG IgM antibodies in sera obtained from mice infected with oocysts peaked significantly at days 1, 10, and 15 (*P*<0.01). The *anti-*BAG1 IgM antibodies in sera obtained from mice infected with tissue cysts peaked significantly at days 15, 40, and 120 (*P*<0.05). The *anti-*GRA1 IgM antibodies in sera obtained from mice infected with oocysts peaked significantly at days 2, 10, and 40 (*P*<0.01). The *anti-*GRA1 IgM antibodies in sera obtained from mice infected with tissue cysts peaked significantly only at day 40 (*P*<0.05). The *anti-*SporoSAG, *anti-*BAG1, and *anti-*GRA1 IgG titers of mice showed significant increases at day 40 (*P*<0.05) and decrement started for only *anti-*GRA1 IgG at day 120. The presence of *anti-*SporoSAG IgM and IgG antibodies can be interpreted as recently acute infection between days 10–40 because IgM decreases at day 40. Similarly, presence of *anti-*BAG1 IgM and absence of IgG can be evaluated as a recently acute infection that occurred 40 days before because IgG peaks at day 40. A peak in *anti-*GRA1 antibody level at first testing and reduction in consecutive sample can be considered as an infection approximately around day 40 or prior. Overall, recombinant SporoSAG, BAG1 and GRA1 proteins can be accepted as valuable diagnostic markers of recently acute toxoplasmosis.

## Introduction

*Toxoplasma gondii* is a medically important parasite that causes congenital toxoplasmosis which manifests as birth defects in unborn children when a seronegative mother is infected during pregnancy [1], [2]. There is not any drug that can affect all the clinical presentations of the illness and the present drugs have teratogenic side effects. Thus, definitive diagnosis of toxoplasmosis has utmost importance for pregnant women. The common approach for diagnosing toxoplasmosis is by serological assays mainly using *T. gondii* tachyzoite lysate antigen. Determining the initiation time of infection that may have occurred in previous 3–4 months (i.e. recently acute infection) is very important for pregnant women who have not been screened for toxoplasmosis before pregnancy. The results of several serological assays are being evaluated together to resolve this issue. However, current commercial or *in house* serological kits still present drawbacks in determining the initiation time of infection.

After 1990s, recombinant protein using ELISA (Rec-ELISA) methods have been developed to diagnose recently acute toxoplasmosis. These studies addressed diagnostic properties of several randomly selected antigenic proteins from surface related proteins (SRS family) as well as rhopty, dense granule, microneme proteins and others [3]–[25]. These studies used well characterized human serum samples but estimation the exact initiation time of infection was not as successful as required.

Diagnosis of recently acute infection may be improved by using stage specific antigens as well as thoroughly collected serum samples such as sera obtained from the systematic follow-up of seroconverted pregnant women in these Rec-ELISAs. Another solution to preliminary validation of these Rec-ELISAs may be the utilization of systematically collected sera obtained from experimentally infected animals. Moreover, antigens specific to sporozoite and bradyzoite forms of the parasite can be used to predict the initiation time of infection since these antigens are no longer expressed by the parasite during tachyzoite form and follow-up of the increment and decrement of antibodies specific to these antigens can point the stage of infection.

Thus, in the present study, “SporoSAG” protein expressed on the surface of sporozoites and “BAG1” protein expressed by bradyzoites were selected as antigens to be used in Rec-ELISAs. In addition, a previously used marker “GRA1” protein expressed by sporozoites, tachyzoites as well as early stage bradyzoites [26]–[34] was also included to the study to compare our results with previous studies and validate the quality of infected animal sera.

Regarding the systematically collected animal sera, two groups of *Swiss outbred* mice were administered orally with fresh sporulated oocysts (contains sporozoites) and tissue cyst to mimic natural route of infection. Serum samples were collected from each mouse prior to infection (day 0) and 1, 2, 3, 6, 10, 15, 40, and 120 days after infection. Thereafter, the kinetics of the specific antibodies against SporoSAG, BAG1, and GRA1 protein, , were followed up by Rec-ELISAs.

## Materials and Methods

### 1. Ethics Statement

All experiments were performed under the instructions and approval of the Institutional Animal Care and Use Committee (IACUC) of Ege University for animal ethical norms (Permit number: 2009–155). Animals were housed under standard and suitable conditions. 6–8 week old female *Swiss outbred* mice were obtained from the Bornova Veterinary Control Institute Animal Production Facility and used during the experiments. To obtain oocysts, recently weaned approximately 3–4 months old kitten was used. The owner of the cat gave permission to be used in the study. The feces of the cat were examined for the presence of oocysts before the experiments, using sucrose flotation technique as described [35].

### 2. Obtaining tissue cysts and oocyts

*T. gondii* PRU strain tissue cysts obtained from mouse brain were fed to cat as described [35]. After feeding the cat, the feces were collected every day and oocysts were purified as described [35]–[37]. Briefly, collected feces (∼10 gr) were added to 50 ml tubes, filled with tap water and incubated for 2 hours at room temperature. Then, tap water was discarded and softened feces was added slowly to approximately 50 ml sucrose solution (53 gr sucrose, 100 ml water, 0,8 ml liquid phenol) and emulsified. Next, the mixture was filtered through two layers of gauze and centrifuged at 400×g for 10 minutes. Thereafter, 0,5 ml supernatant from the top of each tube was collected and mixed with 4,5 ml 2% H_2_SO_4_. The oocysts were incubated at room temperature for 3–5 days. As the oocysts sporulated, 3 ml 1 N NaOH was added.

To purify the oocysts, 4 ml 2.2 M sucrose solution was mixed to each tube. Then, 5 ml distilled H_2_O was slowly added on top of mixture and centrifuged at 1200×g for 20 minutes. Supernatant was collected without touching the sucrose solution. Thereafter, 4 ml 2.2 M sucrose solution was mixed to the supernatant and 5 ml distilled H_2_O was slowly added on top of mixture. After centrifugation at 1200×g for 20 minutes, supernatant was collected and filled up to 50 ml with distilled water. The mixture was centrifuged at 2000×g for 10 minutes and 1–2 ml supernatant collected from the top of the tube was mixed with equal amount of 0,9 % NaCl. The resulting purified oocysts were immediately used to infect mice.

### 3. Infection and collection of mouse sera

Two groups (each contains 6 animals) of Swiss mice were administered with fresh 8–10 sporulated oocysts and 10–15 tissue cysts orally using a stainless steel curved feeding needle (Harvard Apparatus) to mimic natural route of infection. Serum samples were collected from each anesthetized mouse prior to infection (Day 0) and 1, 2, 3, 6, 10, 15, 40, and 120 days after infection. At the end of day 120, mice brains were homogenized in approximately 2 ml sterile 0,9 % NaCl containing Penicillin (10 U/ml), Streptomycin (10 µg/ml) and Gentamicin (2 µg/ml) using an injector with 20G 1″ (0,9×25 mm) needle. Thereafter, the tissue cysts were counted under phase contrast microscopy.

### 4. Cloning of SporoSAG, BAG1, and GRA1 genes into bacterial expression vector

T7 promoter containing pET28a expression vector (10 µg; 5.3 kb; kanamycin resistant; Novagen, USA) was linearized by BamHI as described [38]. The following primers were designed to generate a linear acceptor vector with sequences suitable for cloning by homologous recombination, 5′-GTCGACAAGCTTGCGGCCGCACTCGAGCACCAC-3′ (forward primer, 33 nt) and 5′-CAGCAAATGGGTCGCGGATCCGAATTCGAGCTC-3′ (reverse primer, 33 nt). The linear acceptor vector was amplified as described [38]. The resulting linear pET28a vector was used during *in vivo* recombination cloning.

#### 4.1. PCR amplification of SporoSAG, BAG1, and GRA1 genes

The SporoSAG (GENBANK database accession number AY492338), BAG1 (GENBANK database accession number X82213), and GRA1 from amino acid positions 25–190, excluding the signal peptide (GENBANK database accession number M26007) were isolated from *T. gondii* genomic DNA with primers designed to incorporate adapter termini into the PCR product to facilitate directional cloning by homologous recombination (‘HiRec’) into the pET28a vector. The primers for SporoSAG gene were 5’-CAGCAAATGGGTCGCGGATCCGAATTCGAGCTCTCGCTTCTTAGCCGAGTAGC-3′ (forward primer, 53 nt; overlapping sequences, underlined) 3′-CAGGGTTGGTGTACATGGCTGTCGACAAGCTTGCGGCCGCACTCGAGCACCAC-5′ (reverse primer, 53 nt; overlapping sequences, underlined). The primers for BAG1 were 5′-CAGCAAATGGGTCGCGGATCCGAATTCGAGCTCTCGCTTCTTAGCCGAGTAGC-3′ (forward primer, 53 nt; overlapping sequences, underlined) 5′-CAGGGTTGGTGTACATGGCTGTCGACAAGCTTGCGGCCGCACTCGAGCACCAC-3′ (reverse primer, 53 nt; overlapping sequences, underlined). The primers for GRA1 were 5′-CAGCAAATGGGTCGCGGATCCGAATTCGAGCTCATGGCCGAAGGCGGCGACAACCA-3′ (forward primer, 56 nt; overlapping sequences, underlined) 5′-TCCTAACAGGAGAGAGAGAGGTCGACAAGCTTGCGGCCGCACTCGAGCACCAC-3′ (reverse primer, 53 nt; overlapping sequences, underlined).

SporoSAG, BAG1, and GRA1 genes were isolated from genomic DNA (1–10 ng) using the designed primers (0.5 µM each), 2U of Taq DNA polymerase (Fermentas, USA), 150 µM dNTPs and 1×Taq DNA polymerase reaction buffer with the following calculated PCR protocol; 5 min initial denaturation step at 95°C, followed by 30 cycles of 0.5 min at 95°C, 0.5 min at 50°C, and 3.5 min at 72°C, and a final extension of 10 min at 72°C. The PCR products were visualized by agarose gel electrophoresis, purified using a PCR purification kit (Qiagen, USA) according to the manufacturer’s protocol, and quantitated by spectrophotometry.

#### 4.2. *In vivo* recombination cloning method

*In vivo* recombination cloning was performed as described [38]. Briefly, linear pET28a vector, purified PCR product and DH5α cells (Invitrogen, USA) were mixed and heat shocked. Plasmids from overnight culture were purified using plasmid purification kit according to the manufacturer’s protocol (Qiagen), visualized by agarose gel electrophoresis and sequenced. The resulting plasmids containing SporoSAG, BAG1, and GRA1 genes were named pET28a/SporoSAG, pET28a/BAG1, and pET28a/GRA1, respectively.

### 5. Protein expression and purification

*E. coli* BL21 (DE3) chemically competent cells (Invitrogen) were transformed with pET28a/SporoSAG, pET28a/BAG1, and pET28a/GRA1 plasmids, and grown in 1 liter LB supplemented with 50 µg/ml kanamycin and 50 µg/ml chloramphenicol with vigorous shaking at 37°C up to an optical density of 0.4, calculated at 600 nm. Then, the cells were induced with isopropyl-β-D-thiogalactopyranoside (IPTG) to a final concentration of 0.5 mM with vigorous shaking at 37°C for 4 hours. The cells were centrifuged at 5000×g and the pellet was resuspended with prechilled loading buffer (50 mM Tris-Cl, pH: 7.5, 0.3 M NaCl). Next, the cells were disrupted with an M-110L microfluidizer processor (Microfluidics, USA) at low temperature under internal pressure of 18000 psi. The processed sample was centrifuged at 30000×g for ½ hours at 4°C and clarified supernatant was filtered through a 0.45 µm filter (Corning, USA).

Purification of the protein was performed by ÄKTA FPLC, a liquid chromatography system, which is controlled by UNICORN software, using a 5 ml HiTrap Chelating HP column (GE Health, USA). Clarified filtered supernatant was applied to the HiTrap column with loading buffer. Then, the column was washed with 150 mM imidazole containing 50 mM Tris-Cl, pH 7.5, 0.3 M NaCl buffer. The recombinant proteins were eluted by raising the imidazole concentration to 300 mM. The protein picks were detected by UV280, shown on 12% sodium dodecyl sulfate-polyacrylamide gel (SDS-PAGE) and fractions were concentrated with a Vivaspin filter unit (Sartorius, Germany) at 4°C. The recombinant proteins were further purified with a Superdex 200 gel filtration column (GE Health, USA). Thereafter, the protein picks detected by UV280 were concentrated and quantitated by Bradford method using Comassie blue protein assay reagent (Pierce, USA) and spectrophotometry. Serially diluted serum bovine albumin (BSA) was used as the reference. Resulting recombinant proteins were named as rSporoSAG, rBAG1, and rGRA1.

### 6. SDS-PAGE and Western blotting

Purified recombinant proteins were separated by 12% SDS-PAGE and transferred to a polyvinylidene difluoride (PVDF) transfer membrane (Immobilon-P, Millipore, USA) and blocked by 0.25% casein containing 1xTBS-T buffer (Tris buffered saline containing Tween 20; 20 mM Tris-Cl pH: 7.8, 0.5 M NaCl, 0.5% Tween 20) for 30 minutes. The membranes were probed with a 1∶50 dilution of monoclonal *anti-*polyhistidine antibody (Sigma, Germany) for 1.5 hours. Next, the membranes were washed thrice with 1xTBS-T and probed with a 1∶2500 dilution of alkaline phosphatase-conjugated goat *anti-*mouse IgG (H+L) antibody (Sigma) in 1xTBS. Thereafter, the membranes were washed thrice with 1xTBS-T and 1xTBS and the blot was developed in diethanolamine buffer (10% Diethanolamine, 0.5 mM MgCl2•6H2O, pH: 9.8) containing 4.3% 5-bromo-4-chloro-3-indolyl phosphate (diluted in dimethylacetamide), 4.1% Nitro-BT (diluted in 70% (v/v) dimethylformamide) (Applichem, Germany).

### 7. Rec-ELISA

Each well of nickel chelated plates (Nunc, USA) were washed thrice with 300 µl PBS-T [PBS (pH 7.3) containing 0.05% (v/v) Tween 20] and coated overnight at 4°C with 100 µl purified recombinant protein (concentration of each recombinant protein was 5 µg/ml) diluted in 0.01 M KCl. Next day, plates were washed and blocked (5% nonfat dry milk containing 0.05% PBS-T) for 30 min. Meanwhile, 1/50 and 1/100 dilutions (for the detection of IgM and IgG antibodies, respectively) of mice sera were incubated in blocking buffer supplemented with *E. coli* lysate at a final concentration of 10 mg/ml protein to block *anti-E. coli* antibodies, for 30 minutes. Then, the plates were probed with blocked sera in duplicate for 2 hours at 37°C. After incubation, the plates were washed and probed with peroxidase conjugated *anti-*mouse IgM (1∶2500; Santa-Cruz, USA) and IgG (1∶5000; Sigma) diluted in PBS-T for 1 hour at 37°C. Next, plates were washed and bound antibodies were visualized after adding 3, 3′, 5, 5′ tetramethylbenzidine (TMB) substrate. Reaction was stopped by adding 75 µl of 2 N sulfuric acid and the results were evaluated in a micro titer plate reader (Bio-Tek ELx808, USA) at 450 nm.

Negative control serum samples are the “day 0” serum samples of mice. When antibody response against BAG1 protein is evaluated, “day 0” sera obtained from tissue cyst infected mice are used. Similarly, “day 0” sera obtained from oocyst infected mice were used as negative control in SporoSAG using Rec-ELISA. In Rec-ELISAs using GRA1, “day 0” sera obtained from both oocyst and tissue cyst infected mice. Samples were considered positive if the absorbance value (AV) of the serum sample exceeded the mean AV+2 S.D. of the negative control serum samples ( = cut-off level).

Each plate contained *anti-*polyhistidine antibody (1∶2500, Sigma) probed control wells to determine the presence of His-tagged protein.

Rec-ELISAs, detecting the presence of *anti-*SporoSAG, *anti-*BAG1, and *anti-*GRA1 IgM/IgG antibodies were probed with sera obtained from mice infected orally with fresh oocysts or tissue cysts. As the SporoSAG protein is specific to sporozoite form of the parasite, rSporoSAG-ELISA was probed with mice sera infected with oocysts. Similarly, BAG1 is specific to bradyzoite form of the parasite and rBAG1-ELISA was probed with mice sera infected with tissue cysts. GRA1 is expressed by both form of the parasite and rGRA1-ELISA was probed with mice sera infected with oocysts and tissue cysts. As control, rSporoSAG and rBAG1 coated plates were probed with pooled sera obtained from mice infected orally with tissue cysts and oocysts, respectively.

### 8. Statistical analysis

Data obtained during the study were processed using Prism 3.03 (GraphPad, USA). A two-tailed unpaired *t* test with 95% confidence interval was used to determine the significance between the results of assays. Absorbance values observed from the Rec-ELISA were expressed as mean ± standard deviation (S.D.).

## Results

### 1. Protein expression and purification

**Figure 1:**
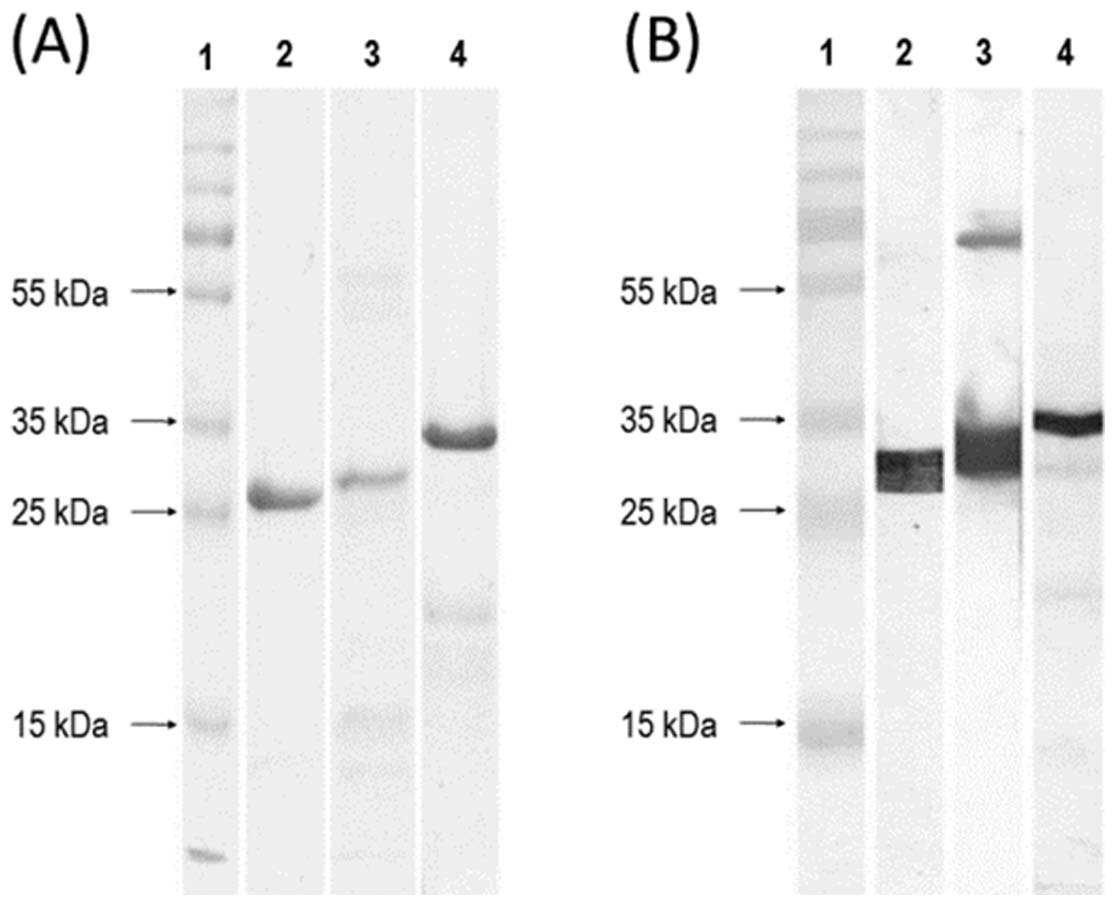
SDS-PAGE (A) and Western blot (B) images of purified rGRA1 (lane 2), rBAG1 (lane 3), and rSporoSAG (lane 4) proteins. Ladder is from Fermentas.

The expression of rSporoSAG, rBAG1, and rGRA1 were induced by 0.5 mM IPTG when the cells grow to an optical density of 0.4 at 600 nm. The cells were harvested after 4 hours, lysed and recombinant proteins were purified by Ni^+2^ chelating column and polished using gel filtration column. The purity of protein product was determined by SDS-PAGE (Figure 1A) and Western blot using *anti-*polyhistidine antibody (Figure 1B). The purified rGRA1, rBAG1, and rSporoSAG expressed from their ORFs’ (GRA1 ORF 663 bp, BAG1 ORF: 846 bp, and SporoSAG ORF: 1035 bp) have calculated molecular masses of 36.06 kDa, 30.71 kDa, and 23.78 kDa, respectively.

### 2. Rec-ELISA

During Rec-ELISA, *anti-*SporoSAG, *anti-*BAG1, and *anti-*GRA1 specific IgM and IgG antibody kinetics were analyzed in sera obtained from two groups of mice administered orally with oocysts and tissue cysts. Serum samples were collected from each mouse prior to infection (Day 0) and 1, 2, 3, 6, 10, 15, 40, and 120 days after infection. At the end of day 120, the presence of tissue cysts were confirmed by microscopy in each mice brain indicative of chronic toxoplasmosis.

#### 2.1. IgM kinetics

**Figure 2:**
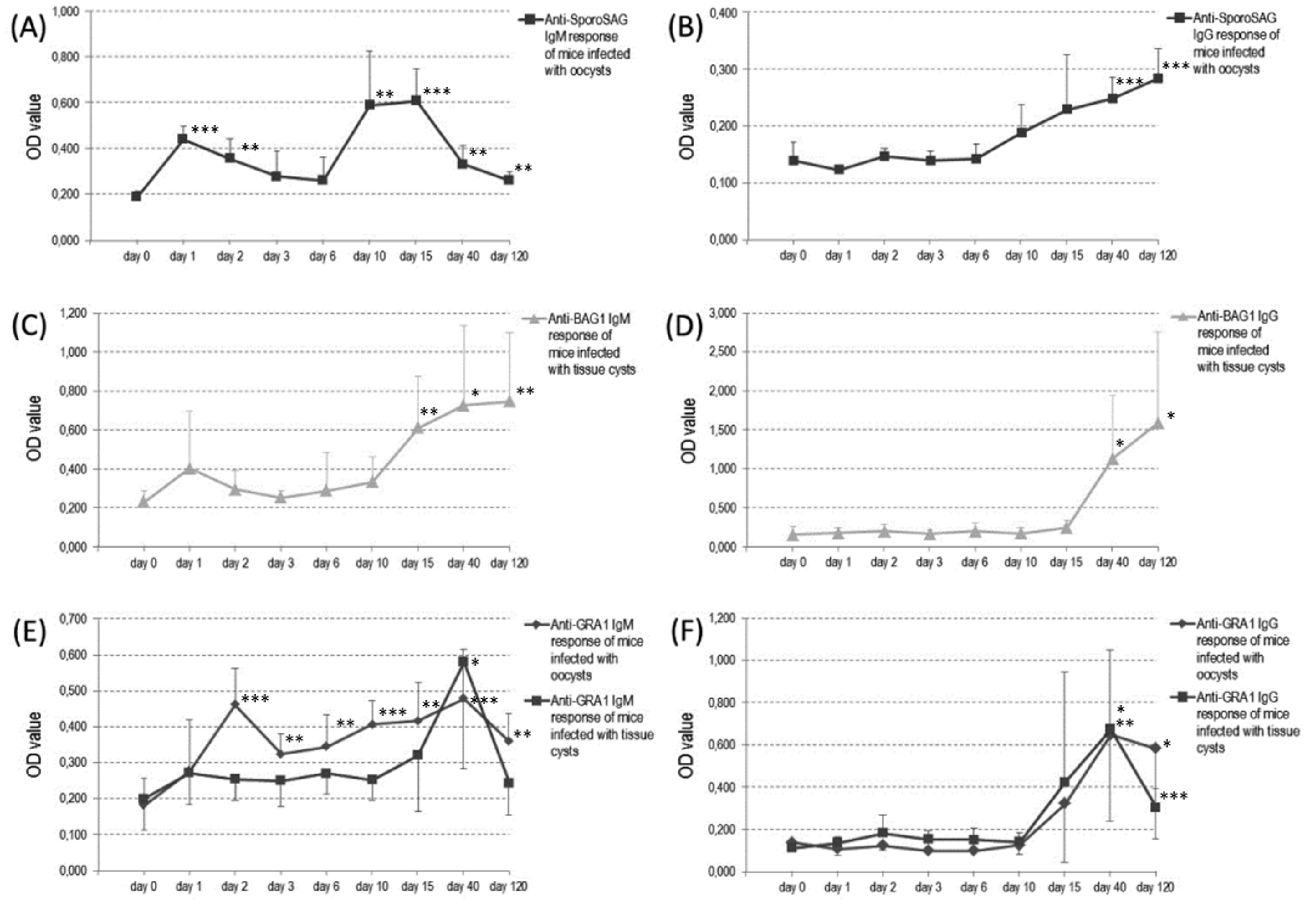
Results of Rec-ELISA using rSporoSAG, rBAG1, and rGRA1 proteins probed with mouse sera. *Anti-*SporoSAG IgM (**A**) and IgG (**B**) responses of mice infected with oocysts. *Anti-*BAG1 IgM (**C**) and IgG (**D**) responses of mice infected with tissue cysts. *Anti-*GRA IgM (**D**) and IgG (**E**) responses of mice infected with oocysts and tissue cysts. Significant increase or decrease in the mean AV is represented by (*), (**), and (***) for *P*<0.05, *P*<0.01, and *P*<0.001 respectively. Each mean AV is derived from sera of 6 mice. Sera have been collected from each mouse prior to infection and 1, 2, 3, 6, 10, 15, 40, and 120 days after infection.

The *anti-*SporoSAG IgM antibodies in sera obtained from mice infected with oocysts peaked significantly at days 1, 10, and 15 compared to day 0 (*P*<0.01) (Figure 2A). After day 1, a decrement is observed and continued until day 6 however increment started afterwards. Thereafter, IgM antibody level dramatically decreased at day 40 sera (Figure 2A).

The *anti-*BAG1 IgM antibodies in sera obtained from mice infected with tissue cysts peaked significantly at days 15, 40, and 120 compared to day 0 (*P*<0.05) (Figure 2C). After day 10, an increment started and continued until day 40. Thereafter, IgM antibody level has almost reached a plateau at day 120 indicative of antibody decrease in the forthcoming days (Figure 2C).

The *anti-*GRA1 IgM antibodies in sera obtained from mice infected with oocysts peaked significantly at days 2, 10, and 40 compared to day 0 (*P*<0.001). After day 2, a decrement is observed for one day however increment started afterwards until day 40 (Figure 2E). The *anti-*GRA1 IgM antibodies in sera obtained from mice infected with tissue cysts peaked significantly only at day 40 compared to day 0 (*P*<0.05). The IgM levels remained at a plateau until day 10 and increment started thereafter until day 40. The IgM antibody levels of both groups decreased after day 40 (Figure 2E).

#### 2.2. IgG kinetics

The *anti-*SporoSAG IgG antibodies in sera obtained from mice infected with oocysts peaked significantly at days 40 and 120 compared to day 0 (*P*<0.001) (Figure 2B). After day 6, an increment started and continued thereafter (Figure 2B). The *anti-*BAG1 IgG antibodies in sera obtained from mice infected with tissue cysts peaked significantly at days 40 and 120 compared to day 0 (*P*<0.05) (Figure 2D). After day 15, an increment started and continued thereafter (Figure 2D).

The *anti-*GRA1 IgG antibodies in sera obtained from mice infected with oocysts peaked significantly at days 40 and 120 compared to day 0 (*P*<0.05) (Figure 2F). After day 10, an increment started which continued until day 40 and thereafter the IgG level did not increase, possibly reached a plateau (Figure 2F). Similarly, the *anti-*GRA1 IgG antibodies in sera obtained from mice infected with tissue cysts peaked significantly at days 40 and 120 compared to day 0 (*P*<0.05) (Figure 2F). After day 10, an increment started which continued until day 40 and thereafter the IgG level started to decrease (Figure 2F).

Interestingly, *anti-*GRA1 IgG antibody levels in sera obtained from mice infected with tissue cysts decreased after day 40, similar to *anti-*GRA1 IgM antibodies (Figure 2E and 2F). The decrement intensity in tissue cyst infected mice was higher than oocyst infected mice in both IgM and IgG responses (Figure 2E and 2F).

The mean absorbance value of each His-tagged protein coated well which is probed with *anti-*polyhistidine antibody was above cut-off level. In addition, the mean absorbance values of rSporoSAG and rBAG1 coated wells which are probed with day 0 pooled sera of mice infected with tissue cysts and oocysts remained below cut-off level. Regarding the day 120 sera, the mean absorbance values of rSporoSAG coated wells probed for IgM and IgG antibody did not exceed the mean AV+2 S.D. (cut-off value) and mean AV+3 S.D. of negative control sera, respectively. The mean absorbance values of rBAG1 coated wells probed for IgM and IgG antibody did not exceed the mean AV+3 S.D. of negative control sera.

## Discussion

Currently, Rec-ELISA methods using different antigenic proteins are being evaluated to diagnose recently acute toxoplasmosis. Well categorized human sera are being used to assess the diagnostic value of these markers [4]. These studies have addressed diagnostic values of several antigenic proteins from SRS family (SAG1, SAG2, SAG2A) rhopty (ROP1, ROP2) dense granules (GRA1, GRA2, GRA3, GRA4, GRA5, GRA6, GRA7, GRA8), microneme (MIC1, MIC2, MIC3, MIC4) and others M2AP, AMA1, HSP20, BAG1 (HSP30), MAG1, NTPase, P25, P35, P68 as well as a multi-epitope peptide (containing epitopes from SAG1, SAG2 and SAG3 proteins) [3]–[25].

These studies evidenced two bottlenecks, in particular, whether the selected antigens have enough specificity, and whether serum samples are collected systematically. *T. gondii* is acquired naturally through ingestion of tissue cysts (contains bradyzoites) or oocysts (contains sporozoites). After the ingestion of oocysts or tissue cysts, sporozoites and bradyzoites are released, invade the intestinal cells and turn in to tachyzoites in 12 and 18 hours, respectively [39], [40]. During natural course of infection, immune response forms against the antigenic proteins of sporozoites and bradyzoites initially and then to tachyzoite form. Therefore, sporozoite and bradyzoite specific immune response have importance for the diagnosis of recently acute toxoplasmosis patients. In addition, antigens expressed by all forms of the parasite can be prioritized.

In the present study, the diagnostic values of SporoSAG (sporozoite specific protein), BAG1 (bradyzoite specific protein), and GRA1 (specific for all forms of the parasite) have been evaluated. SporoSAG is from the SRS family proteins of *T. gondii* and has attachment function for host cell invasion. In addition, it is the most abundantly expressed protein on the infectious sporozoite surface as shown by transcriptomic and proteomic analysis conducted on oocysts/sporozoites [26]–[29]. Overall, SporoSAG protein appears to be the most convenient protein to monitor the antibody response to sporozoite originated infection.

BAG1 is a bradyzoite specific cytoplasmic antigen which has similarities to small heat shock proteins (HSPs) of plants [30]. The expression of BAG1 is upregulated in bradyzoites as shown by transcriptomic analyses [30], [31]. It has been reported that BAG1 facilitates the tissue cyst formation and takes important roles in the formation of immune response against toxoplasmosis [22], [30], [31]. These properties make BAG1 an appropriate protein to observe antibody response to tissue cyst originated infection.

GRA1 is located in the electron dense secretory organelles, which continuously release their content into the parasitophorous vacuole (PV) during the intracellular development of tachyzoites. GRA1 functions as a calcium-binding protein and is found in a soluble form in the lumen and network of the PV during invasion of the host cell [32], [33]. GRA1 has shown to be up regulated in fully sporulated oocysts [27], [34]. Although GRA1 is being expressed at the beginning of conversion from tachyzoites to bradyzoites, its expression is progressively repressed after the second day of conversion process [31]. Altogether, GRA1 seems to be an ideal protein to determine the course of antibody response to all forms of the parasite.

Secondly, the probable initiation time of toxoplasmosis in humans cannot be properly determined because the clinical symptoms appear in approximately 10% of the patients and besides symptomatic patients give subtly flu like findings. Moreover, serum samples of recently acute infected patients are not always systematically collected which makes the evaluation of these assays even more difficult. The type of human sera that can help validate these assays can be sera collected from the systematic follow-up of seroconverted pregnant women as well as outbreak originated serum samples. Unfortunately, it is hard to detect an outbreak worldwide due to covert clinical findings.

An alternative to human sera is the serum samples obtained from animals experimentally infected through natural route of infection which can validate the diagnostic value of these antigens in terms of determining the initiation time of infection. In the present study, sera obtained from a murine model infected orally (to mimic natural route of infection) with fresh oocysts and tissue cysts were used to evaluate the Rec-ELISAs.

During the course of humoral immune response, IgM antibodies, which are the most valuable markers of recently acute infection, become evident at the first days of infection. IgG antibodies persist for the lifetime of the individual while IgM antibodies rapidly increase and subsequently, decline or disappear at highly variable rates over several years [41]. Chardes et al. investigated antibody responses in serum of mice orally infected with *T. gondii* tissue cysts by ELISA using *T. gondii* tachyzoite lysate. They found that IgG antibodies against *T. gondii* were first detected on day 14, reached a plateau on day 28, and remained at the same level throughout the rest of the research. In contrast, the titers of IgM antibodies against *T. gondii* peaked on day 14 and then decreased progressively [42]. These results are comparable with antibody response to GRA1 protein induced by tissue cyst/oocyst infection in the present study.

Gatkowska et al. evaluated the usefulness of GRA1, GRA6, GRA7, p35, SAG1, SAG2, ROP2, and ROP4 using ELISA as a diagnostic marker for recently acute toxoplasmosis in sera obtained from mice inoculated intraperitoneally with *T. gondii* DX strain tissue cysts in two distinct studies [43], [44]. In their first study, sera were collected at days 7, 21, and 56 after infection. According to the results, GRA6, GRA7 and p35 were highly responsive during acute infection whereas strong reactivity against GRA1, SAG1 and SAG2 were observed during chronic infection. The IgM+IgG arithmetic mean of *anti-*GRA1 was below cut-off at day 7 of infection and above cut-off level 21 days after infection, which are comparable with the present study. The IgM+IgG response to GRA1 showed increment at day 56 of infection. In the present study, *anti-*GRA1 IgM and IgG titers reached to their highest levels at day 40 and then decreased at day 120 of infection (Figure 2E and 2F). The decrease in GRA1 levels after 40 days of infection may be due to low expression of GRA1 by bradyzoites during tissue cyst formation [31]. Based on these data, the *anti-*GRA1 levels can give an idea about the time of infection. For example, the time of infection in a patient giving a peak *anti-*GRA1 antibody level at first testing and reduction in consecutive sample, can be considered as approximately around day 40 or before during first testing.

The efficacy of GRA1 in detecting recently acute infection has been evaluated by human sera also [4], [13], [14], [17]. Lecordier et al. compared the *anti-*GRA1 IgG and *anti-*GRA6 IgG levels of human samples and stated that the sensitivity of GRA1 IgG ELISA was low (68%) compared to GRA6 IgG ELISA (96%). The sera used in this study were characterized based on IgG level and recently acute cases were not sorted as another group [13]. Pietkiewicz et al. evaluated the diagnostic value of GRA1 as well as GRA7 and SAG1 in recently acute sera. They stated that the least reactive recombinant protein was GRA1 (83.3%) compared to GRA7 (95.9%) and SAG1 (98.6%) [17]. The *anti-*GRA1 seronegative patients leading to low sensitivity may be after day 120 of the infection as shown in the present study and moreover infected with tissue cysts (the level of IgG in tissue infected mice decreases more rapidly) (Figure 2F). Ferrandiz et al. further evaluated the diagnostic efficacy of GRA1 and GRA6 in recently acute serum samples (seroconversion within 3 months) and in some follow up sera of seroconverted patients [14]. The 34% and 84% of recently acute samples reacted with GRA1 and GRA6, respectively. Among the follow up sera, three samples were non-responsive to GRA1 for 112 days and three samples started to give response at day 84. Interestingly, the remaining four samples started to give response at day 28, peaked at day 49 and thereafter progressively decreased which is very similar to the *anti-*GRA1 IgG titer curve observed in this study (Figure 2F). Altogether, GRA1 appears to be a good marker as *T. gondii* lysate antigen to monitor the acute and chronic phases of infection.

The diagnostic value of BAG1 protein has been evaluated with sera from patients whom the time of seroconversion was known and IgG antibodies reacted with BAG1 protein as early as one month after infection in these samples. Overall, it has been stated that human sera reacts with BAG1 protein promptly after acute infection [22]. In the present study, *anti-*BAG1 IgG and IgM response to BAG1 protein has been evaluated first time in murine infected orally with tissue cysts and oocysts. Similarly, the *anti-*BAG1 IgM antibodies peaked significantly at day 15 (*P*<0.01) and reached a plateau at day 120 (Figure 2C). The *anti-*BAG1 IgG antibody levels peaked significantly increase at day 40 and increment continued until day 120 (*P*<0.05) (Figure 2D). These results show that BAG1 is a powerful antigen to detect antibody response during tissue cyst related acute and chronic phases of infection.

Crawford et al. evaluated the diagnostic value of SporoSAG protein using recently acute infection sera from humans (possibly oocyst infected) as well as with one mouse serum sample infected orally with sporulated oocysts. Although SporoSAG has been shown to be the most abundantly expressed protein on the infectious sporozoite surface as shown by transcriptomic and proteomic analysis, IgM, IgG or IgA response specific to SporoSAG protein was not detected in human and mouse sera [28], [29], [45]. In the present study, the IgM and IgG levels of *anti-*SporoSAG antibodies were screened for 4 months in consecutive serum samples obtained from 6 mice infected orally with oocysts. The IgM antibodies peaked significantly at day 10 and drastically decreased at day 40 (Figure 2A). The IgG antibody level significantly increased at day 40 and increment continued until day 120 (*P*<0.001) (Figure 2B). The IgG signal intensity of SporoSAG is weak compared to GRA1 and BAG1 however consecutive serum sampling shows the increment of IgG response to SporoSAG protein during the course of acute infection. These results show that follow-up of antibody response against SporoSAG protein can elucidate the mist over sporozoite originated acute infection.

Overall, recombinant SporoSAG, BAG1, and GRA1 proteins can be accepted as valuable diagnostic markers of recently acute toxoplasmosis in murine model. Future studies should evaluate peak levels of IgM, IgA and IgG antibodies to more stage specific antigens of *T. gondii* comprehensively using different animal models as well as sera obtained from the systematic follow-up of seroconverted pregnant women and outbreak samples to generate a multi-recombinant ELISA to predict the initiation time of recently acute infection in humans.
